# Supplementary material for: Prolonged decay of molecular rate estimates for metazoan mitochondrial DNA
Source: PeerJ. 2015 Mar 5;3:e821. doi: 10.7717/peerj.821 (PMC4358697; doi:10.7717/peerj.821)
Supplement: Supplemental Information 1 — Intraspecific time-dependent rates—human case study; and Supplementary material references. [file peerj-03-821-s001.pdf]

Prolonged decay of molecular rate estimates for metazoan mitochondrial DNA

Martyna Molak and Simon Y. W. Ho

Supplementary material

Intraspecific time-dependent rates – human case study

A separate regression analysis was performed on human mitochondrial rates against their calibration times to test for time-dependent rates at the intraspecific level. Humans were the only taxon for which there were a large number of rate estimates for a wide range of calibration times. This case study included 26 estimates of rates in the human mitochondrial control region. In the interspecific regressions, we had pooled these estimates into only a few data points. Calibrations were based on various sources of information and ranged from 25 years (one generation) to 12.5 Myr (maximum age for the *Homo*-*Pongo* split). Rate estimates ranged from 1.33×10^{-8} substitutions/site/year (phylogenetic estimate calibrated using the *Pan*-*Homo* split; Pesole *et al.* 1999) to 2.69×10^{-6} mutations/site/year (pedigree study of a Costa Rican population; Madrigal *et al.* 2012). Regression analysis produced strong evidence of a negative relationship between rate estimates and calibration times (slope -0.24, $p = 1.51\times10^{-7}$, $R^2 = 0.69$; Figure S1, Table S4).

Supplementary material references:

Allegrucci G, Trucchi E, and Sbordoni V. 2011. Tempo and mode of species diversification in Dolichopoda cave crickets (Orthoptera, Rhaphidophoridae). *Molecular Phylogenetics and Evolution* 60:108-121.

Alter SE, Rynes E, and Palumbi SR. 2007. DNA evidence for historic population size and past ecosystem impacts of gray whales. *Proceedings of the National Academy of Sciences of the United States of America* 104:15162-15167.

Andersen NM, Cheng L, Damgaard J, and Sperling FAH. 2000. Mitochondrial DNA sequence variation and phylogeography of oceanic insects (Hemiptera: Gerridae: *Halobates* spp.). *Marine Biology* 136:421-430.

Arbogast BS, Drovetski SV, Curry RL, Boag PT, Seutin G, Grant PR, Grant BR, and Anderson DJ. 2006. The origin and diversification of Galapagos mockingbirds. *Evolution* 60:370-382.

Arensburger P, Buckley TR, Simon C, Moulds M, and Holsinger KE. 2004. Biogeography and phylogeny of the New Zealand cicada genera (Hemiptera: Cicadidae) based on nuclear and mitochondrial DNA data. *Journal of Biogeography* 31:557-569.

Arnold EN, Vasconcelos R, Harris DJ, Mateo JA, and Carranza S. 2008. Systematics, biogeography and evolution of the endemic Hemidactylus geckos (Reptilia, Squamata, Gekkonidae) of the Cape Verde

Islands: based on morphology and mitochondrial and nuclear DNA sequences. *Zoologica Scripta* 37:619-636.

Baker AJ, Pereira SL, Haddrath OP, and Edge KA. 2006. Multiple gene evidence for expansion of extant penguins out of Antarctica due to global cooling. *Proceedings of the Royal Society B - Biological Sciences* 273:11-17.

Baker AJ, Pereira SL, and Paton TA. 2007. Phylogenetic relationships and divergence times of Charadriiformes genera: multigene evidence for the Cretaceous origin of at least 14 clades of shorebirds. *Biology Letters* 3:205-209.

Balke M, Pons J, Ribera I, Sagata K, and Vogler AP. 2007. Infrequent and unidirectional colonization of hyperdiverse Papuadytes diving beetles in New Caledonia and New Guinea. *Molecular Phylogenetics and Evolution* 42:505-516.

Barker FK, Cibois A, Schikler P, Feinstein J, and Cracraft J. 2004. Phylogeny and diversification of the largest avian radiation. *Proceedings of the National Academy of Sciences of the United States of America* 101:11040-11045.

Barnett R, Shapiro B, Barnes I, Ho SYW, Burger J, Yamaguchi N, Higham TF, Wheeler HT, Rosendahl W, Sher AV, Sotnikova M, Kuznetsova T, Baryshnikov GF, Martin LD, Harington CR, Burns JA, and Cooper A. 2009. Phylogeography of lions (*Panthera leo* ssp.) reveals three distinct taxa and a late Pleistocene reduction in genetic diversity. *Molecular Ecology* 18:1668-1677.

Barry JC, Morgan MLE, Flynn LJ, Pilbeam D, Behrensmeyer AK, Raza SM, Khan IA, Badgley C, Hicks J, and Kelley J. 2002. Faunal and environmental change in the late Miocene Siwaliks of northern Pakistan. *Paleobiology* 28:1-71.

Becker J. 1987. *Neogene avian localities of North America*. Washington, D.C.: Smithsonian Institution Press.

Bendall KE, Macaulay VA, Baker JR, and Sykes BC. 1996. Heteroplasmic point mutations in the human mtDNA control region. *American Journal of Human Genetics* 59:1276-1287.

Benz BW, Robbins MB, and Peterson AT. 2006. Evolutionary history of woodpeckers and allies (Aves: Picidae): placing key taxa on the phylogenetic tree. *Molecular Phylogenetics and Evolution* 40:389-399.

Boev ZN, and Koufos GD. 2006. The late Miocene vertebrate locality of Perivolaki, Thessaly, Greece: 2. Aves. *Palaeontographica Abteilung A* 276:11-22.

Bohning-Gaese K, Caprano T, van Ewijk K, and Veith M. 2006. Range size: disentangling current traits and phylogenetic and biogeographic factors. *American Naturalist* 167:555-567.

Bohrson WA, and Reid MR. 1997. Genesis of silicic peralkaline volcanic rocks in an ocean island setting by crustal melting and open-system processes: Socorro Island, Mexico. *Journal of Petrology* 38:1137-1166.

Boles WE. 1998. A Budgerigar *Melopsittacus undulatus* from the Pliocene of Riversleigh, north-western Queensland. *Emu* 98:32-35.

Bollongino R, Burger J, Powell A, Mashkour M, Vigne JD, and Thomas MG. 2012. Modern taurine cattle descended from small number of Near-Eastern founders. *Molecular Biology and Evolution* 29:2101-2104.

Bollongino R, Edwards CJ, Alt KW, Burger J, and Bradley DG. 2006. Early history of European domestic cattle as revealed by ancient DNA. *Biology Letters* 2:155-159.

Bridge ES, Jones AW, and Baker AJ. 2005. A phylogenetic framework for the terns (Sternini) inferred from mtDNA sequences: implications for taxonomy and plumage evolution. *Molecular Phylogenetics and Evolution* 35:459-469.

Brotherton P, Haak W, Templeton J, Brandt G, Soubrier J, Adler CJ, Richards SM, Der Sarkissian C, Ganslmeier R, and Friederich S. 2013. Neolithic mitochondrial haplogroup H genomes and the genetic origins of Europeans. *Nature Communications* 4:1764.

Brower AVZ. 1994. Rapid morphological radiation and convergence among races of the butterfly *Heliconius erato* inferred from patterns of mitochondrial DNA evolution. *Proceedings of the National Academy of Sciences of the United States of America* 91:6491-6495.

Bryson RW, Garcia-Vazquez UO, and Riddle BR. 2012. Relative roles of Neogene vicariance and Quaternary climate change on the historical diversification of bunchgrass lizards (Sceloporus scalaris group) in Mexico. *Molecular Phylogenetics and Evolution* 62:447-457.

Burns KJ, Hackett SJ, and Klein NK. 2002. Phylogenetic relationships and morphological diversity in Darwin's finches and their relatives. *Evolution* 56:1240-1252.

Burns KJ, and Naoki K. 2004. Molecular phylogenetics and biogeography of Neotropical tanagers in the genus Tangara. *Molecular Phylogenetics and Evolution* 32:838-854.

Caccone A, and Sbordoni V. 2001. Molecular biogeography of cave life: a study using mitochondrial DNA from Bathysciine beetles. *Evolution* 55:122-130.

Campos PF, Kristensen T, Orlando L, Sher A, Kholodova MV, Gotherstrom A, Hofreiter M, Drucker DG, Kosintsev P, Tikhonov A, Baryshnikov GF, Willerslev E, and Gilbert MTP. 2010a. Ancient DNA sequences point to a large loss of mitochondrial genetic diversity in the saiga antelope (*Saiga tatarica*) since the Pleistocene. *Molecular Ecology* 19:4863-4875.

Campos PF, Willerslev E, Sher A, Orlando L, Axelsson E, Tikhonov A, Aaris-Sorensen K, Greenwood AD, Kahlke RD, Kosintsev P, Krakhmalnaya T, Kuznetsova T, Lemey P, MacPhee R, Norris CA, Shepherd K, Suchard MA, Zazula GD, Shapiro B, and Gilbert MTP. 2010b. Ancient DNA analyses exclude humans as the driving force behind late Pleistocene musk ox (*Ovibos moschatus*) population dynamics. *Proceedings of the National Academy of Sciences of the United States of America* 107:5675-5680.

Carranza S, Arnold E, and Amat F. 2004. DNA phylogeny of Lacerta (Iberolacerta) and other lacertine lizards (Reptilia: Lacertidae): did competition cause long-term mountain restriction? *Systematics and Biodiversity* 2:57-77.

Carranza S, Arnold EN, Geniez P, Roca J, and Mateo JA. 2008. Radiation, multiple dispersal and parallelism in the skinks, *Chalcides* and *Sphenops* (Squamata: Scincidae), with comments on *Scincus* and *Scincopus* and the age of the Sahara Desert. *Molecular Phylogenetics and Evolution* 46:1071-1094.

Carroll RL. 1988. Ungulates, edentates, and whales. In: Carroll RL, ed. *Vertebrate Paleontology and Evolution*. New York, USA: W. H. Freeman and Co, 502–568.

Carson HL, and Calgue DA. 1995. Geology and biogeography of the Hawaiian Islands. In: Wagner W, and Funk V, eds. *Hawaiian biogeography: evolution in a hotspot archipelago*. Washington, DC: Smithsonian Institution Press, 14-19.

Carson RJ, and Spicer GS. 2003. A phylogenetic analysis of the emberizid sparrows based on three mitochondrial genes. *Molecular Phylogenetics and Evolution* 29:43-57.

Cavelier L, Jazin E, Jalonen P, and Gyllensten U. 2000. mtDNA substitution rate and segregation of heteroplasmy in coding and noncoding regions. *Human Genetics* 107:45-50.

Chan YL, Anderson CN, and Hadly EA. 2006. Bayesian estimation of the timing and severity of a population bottleneck from ancient DNA. *PLOS Genetics* 2:e59.

Chandler RM. 1990. Fossil birds of the San Diego Formation, late Pliocene, Blancan, San Diego County, California, Part 2. *Recent advances in the study of Neogene fossil birds*, 73–171.

Clarke TE, Levin DB, Kavanaugh DH, and Reimchen TE. 2001. Rapid evolution in the *Nebria gregaria* group (Coleoptera: Carabidae) and the paleogeography of the Queen Charlotte Islands. *Evolution* 55:1408-1418.

Coates AG, and Obando JA. 1996. The geologic evolution of the Central American isthmus. In: Jackson JBC, Budd AF, and Coates AG, eds. *Evolution and environment in tropical America*. Chicago, Illinois: University of Chicago Press, 21–56.

Cohen BL, Baker AJ, Bleschschmidt K, Dittmann DL, Furness RW, Gerwin JA, Helbig AJ, DeKorte J, Marshall HD, Palma RL, Peter HU, Ramli R, Siebold I, Willcox MS, Wilson RH, and Zink RM. 1997. Enigmatic phylogeny of skuas (Aves: Stercorariidae). *Proceedings of the Royal Society B - Biological Sciences* 264:181-190.

Colangelo P, Bannikova AA, Krystufek B, Lebedev VS, Annesi F, Capanna E, and Loy A. 2010. Molecular systematics and evolutionary biogeography of the genus Talpa (Soricomorpha: Talpidae). *Molecular Phylogenetics and Evolution* 55:372-380.

Cox SC, Carranza S, and Brown RP. 2010. Divergence times and colonization of the Canary Islands by Gallotia lizards. *Molecular Phylogenetics and Evolution* 56:747-757.

Dalén L, Nyström V, Valdiosera C, Germonpré M, Sablin M, Turner E, Angerbjörn A, Arsuaga JL, and Götherström A. 2007. Ancient DNA reveals lack of postglacial habitat tracking in the arctic fox. *Proceedings of the National Academy of Sciences of the United States of America* 104:6726-6729.

Daza JM, Smith EN, Paez VP, and Parkinson CL. 2009. Complex evolution in the Neotropics: The origin and diversification of the widespread genus Leptodeira (Serpentes: Colubridae). *Molecular Phylogenetics and Evolution* 53:653-667.

Debruyne R, Chu G, King CE, Bos K, Kuch M, Schwarz C, Szpak P, Grocke DR, Matheus P, Zazula G, Guthrie D, Froese D, Buigues B, de Marliave C, Flemming C, Poinar D, Fisher D, Southon J, Tikhonov AN, MacPhee RDE, and Poinar HN. 2008. Out of America: ancient DNA evidence for a new world origin of late quaternary woolly mammoths. *Current Biology* 18:1320-1326.

Desalle R, Freedman T, Prager EM, and Wilson AC. 1987. Tempo and mode of sequence evolution in mitochondrial DNA of Hawaiian *Drosophila*. *Journal of Molecular Evolution* 26:157-164.

Donne-Gousse C, Laudet V, and Hanni C. 2002. A molecular phylogeny of anseriformes based on mitochondrial DNA analysis. *Molecular Phylogenetics and Evolution* 23:339-356.

Dornburg A, Brandley MC, McGowen MR, and Near TJ. 2012. Relaxed clocks and inferences of heterogeneous patterns of nucleotide substitution and divergence time estimates across whales and dolphins (Mammalia: Cetacea). *Molecular Biology and Evolution* 29:721-736.

Duchêne S, Frey A, Alfaro-Nunez A, Dutton PH, Gilbert MTP, and Morin PA. 2012. Marine turtle mitogenome phylogenetics and evolution. *Molecular Phylogenetics and Evolution* 65:241-250.

Edwards CJ, Bollongino R, Scheu A, Chamberlain A, Tresset A, Vigne JD, Baird JF, Larson G, Ho SYW, Heupink TH, Shapiro B, Freeman AR, Thomas MG, Arbogast RM, Arndt B, Bartosiewicz L,

Benecke N, Budja M, Chaix L, Choyke AM, Coqueugniot E, Dohle HJ, Goldner H, Hartz S, Helmer D, Herzig B, Hongo H, Mashkour M, Ozdogan M, Pucher E, Roth G, Schade-Lindig S, Schmolcke U, Schulting RJ, Stephan E, Uerpmann HP, Voros I, Voytek B, Bradley DG, and Burger J. 2007. Mitochondrial DNA analysis shows a Near Eastern Neolithic origin for domestic cattle and no indication of domestication of European aurochs. *Proceedings of the Royal Society B - Biological Sciences* 274:1377-1385.

Emslie SD. 1998. *Avian community, climate, and sea-level changes in the Plio-Pleistocene of the Florida Peninsula*. Washington, D.C.: American Ornithologists' Union.

Emslie SD. 2007. Fossil passerines from the early Pliocene of Kansas and the evolution of songbirds in North America. *The Auk* 124:85-95.

Endicott P, and Ho SYW. 2008. A Bayesian evaluation of human mitochondrial substitution rates. *American Journal of Human Genetics* 82:895-902.

Esseghir S, Ready PD, and Ben-Ismail R. 2000. Speciation of *Phlebotomus* sandflies of the subgenus *Larroussius* coincided with the late Miocene-Pliocene aridification of the Mediterranean subregion. *Biological Journal of the Linnean Society* 70:189-219.

Esseghir S, Ready PD, Killick-Kendrick R, and Ben-Ismail R. 1997. Mitochondrial haplotypes and phylogeography of *Phlebotomus* vectors of *Leishmania major*. *Insect Molecular Biology* 6:211-225.

Farrell BD. 2001. Evolutionary assembly of the milkweed fauna: cytochrome oxidase I and the age of Tetraopes beetles. *Molecular Phylogenetics and Evolution* 18:467-478.

Fleischer RC, McIntosh CE, and Tarr CL. 1998. Evolution on a volcanic conveyor belt: using phylogeographic reconstructions and K-Ar-based ages of the Hawaiian Islands to estimate molecular evolutionary rates. *Molecular Ecology* 7:533-545.

Flynn JJ, and Galiano H. 1982. Phylogeny of early Tertiary Carnivora: with a description of a new species of *Protictis* from the Middle Eocene of northwestern Wyoming. *American Museum Novitates* 2725:1-64.

Fontanella FM, Olave M, Avila LJ, Sites Jr JW, and Morando M. 2012. Molecular dating and diversification of the South American lizard genus Liolaemus (subgenus Eulaemus) based on nuclear and mitochondrial DNA sequences. *Zoological Journal of the Linnean Society* 164:825-835.

Foote AD, Kaschner K, Schultze SE, Garilao C, Ho SYW, Post K, Higham TFG, Stokowska C, van der Es H, Embling CB, Gregersen K, Johansson F, Willerslev E, and Gilbert MTP. 2013. Ancient DNA reveals that bowhead whale lineages survived Late Pleistocene climate change and habitat shifts. *Nature Communications* 4:1677.

Forcina G, Panayides P, Guerrini M, Nardi F, Gupta BK, Mori E, Al-Sheikhly OF, Mansoori J, Khaliq I, Rank DN, Parasharya BM, Khan AA, Hadjigerou P, and Barbanera F. 2012. Molecular evolution of the Asian francolins (Francolinus, Galliformes): A modern reappraisal of a classic study in speciation. *Molecular Phylogenetics and Evolution* 65:523-534.

Friesen VL, Baker AJ, and Piatt JF. 1996. Phylogenetic relationships within the Alcidae (Charadriiformes: Aves) inferred from total molecular evidence. *Molecular Biology and Evolution* 13:359-367.

Fu Q, Mittnik A, Johnson PL, Bos K, Lari M, Bollongino R, Sun C, Giemsch L, Schmitz R, Burger J, Ronchitelli AM, Martini F, Cremonesi RG, Svoboda J, Bauer P, Caramelli D, Castellano S, Reich D, Pääbo S, and Krause J. 2013. A revised timescale for human evolution based on ancient mitochondrial genomes. *Current Biology* 23:553-559.

Fuchs J, Chen S, Johnson JA, and Mindell DP. 2011. Pliocene diversification within the South American Forest falcons (Falconidae: Micrastur). *Molecular Phylogenetics and Evolution* 60:398-407.

García-Moreno J, Cortés N, Garcia-Deras GM, and Hernández-Baños BE. 2006. Local origin and diversification among *Lampornis* hummingbirds: a Mesoamerican taxon. *Molecular Phylogenetics and Evolution* 38:488-498.

Geurgas SR, and Rodrigues MT. 2010. The hidden diversity of *Coleodactylus amazonicus* (Sphaerodactylinae, Gekkota) revealed by molecular data. *Molecular Phylogenetics and Evolution* 54:583-593.

Göhlich UB. 2007. The oldest fossil record of the extant penguin genus *Spheniscus* - a new species from the Miocene of Peru. *Acta Palaeontologica Polonica* 52:285-298.

Goios A, Pereira L, Bogue M, Macaulay V, and Amorim A. 2007. mtDNA phylogeny and evolution of laboratory mouse strains. *Genome Research* 17:293-298.

Gómez-Zurita J, Juan C, and Petitpierre E. 2000. The evolutionary history of the genus Timarcha (Coleoptera, Chrysomelidae) inferred from mitochondrial COII gene and partial 16S rDNA sequences. *Molecular Phylogenetics and Evolution* 14:304-317.

Grafe K, Frisch W, Villa IM, and Meschede M. 2002. Geodynamic evolution of southern Costa Rica related to low-angle subduction of the Cocos Ridge: constraints from thermochronology. *Tectonophysics* 348:187-204.

Gratton P, Konopiński MK, and Sbordoni V. 2008. Pleistocene evolutionary history of the Clouded Apollo (*Parnassius mnemosyne*): genetic signatures of climate cycles and a 'time-dependent' mitochondrial substitution rate. *Molecular Ecology* 17:4248-4262.

Groombridge JJ, Jones CG, Bayes MK, van Zyl AJ, Carrillo J, Nichols RA, and Bruford MW. 2002. A molecular phylogeny of African kestrels with reference to divergence across the Indian Ocean. *Molecular Phylogenetics and Evolution* 25:267-277.

Guo XG, Dai X, Chen DL, Papenfuss TJ, Ananjeva NB, Melnikov DA, and Wang YZ. 2011. Phylogeny and divergence times of some racerunner lizards (Lacertidae: Eremias) inferred from mitochondrial 16S rRNA gene segments. *Molecular Phylogenetics and Evolution* 61:400-412.

Hall ML. 1983. Origin of Espanola-Island and the Age of Terrestrial Life on the Galapagos-Islands. *Science* 221:545-547.

Hasegawa M, and Horai S. 1991. Time of the deepest root for polymorphism in human mitochondrial DNA. *Journal of Molecular Evolution* 32:37-42.

Hay JM, Subramanian S, Millar CD, Mohandesan E, and Lambert DM. 2008. Rapid molecular evolution in a living fossil. *Trends in Genetics* 24:106-109.

Heller R, Bruniche-Olsen A, and Siegismund HR. 2012. Cape buffalo mitogenomics reveals a Holocene shift in the African human-megafauna dynamics. *Molecular Ecology* 21:3947-3959.

Heyer E, Zietkiewicz E, Rochowski A, Yotova V, Puymirat J, and Labuda D. 2001. Phylogenetic and familial estimates of mitochondrial substitution rates: study of control region mutations in deep-rooting pedigrees. *American Journal of Human Genetics* 69:1113-1126.

Ho SYW, and Lanfear R. 2010. Improved characterisation of among-lineage rate variation in cetacean mitogenomes using codon-partitioned relaxed clocks. *Mitochondrial DNA* 21:138-146.

Ho SYW, Larson G, Edwards CJ, Heupink TH, Lakin KE, Holland PWH, and Shapiro B. 2008. Correlating Bayesian date estimates with climatic events and domestication using a bovine case study. *Biology Letters* 4:370-374.

Ho SYW, Phillips MJ, Cooper A, and Drummond AJ. 2005. Time dependency of molecular rate estimates and systematic overestimation of recent divergence times. *Molecular Biology and Evolution* 22:1561-1568.

Hofreiter M, Capelli C, Krings M, Waits L, Conard N, Munzel S, Rabeder G, Nagel D, Paunovic M, Jambresic G, Meyer S, Weiss G, and Pääbo S. 2002. Ancient DNA analyses reveal high mitochondrial DNA sequence diversity and parallel morphological evolution of late pleistocene cave bears. *Molecular Biology and Evolution* 19:1244-1250.

Hope AG, Waltari E, Dokuchaev NE, Abramov S, Dupal T, Tsvetkova A, Henttonen H, MacDonald SO, and Cook JA. 2010. High-latitude diversification within Eurasian least shrews and Alaska tiny shrews (Soricidae). *Journal of Mammalogy* 91:1041-1057.

Horai S, Hayasaka K, Kondo R, Tsugane K, and Takahata N. 1995. Recent African origin of modern humans revealed by complete sequences of hominoid mitochondrial DNAs. *Proceedings of the National Academy of Sciences of the United States of America* 92:532-536.

Howell N, Kubacka I, and Mackey DA. 1996. How rapidly does the human mitochondrial genome evolve? *American Journal of Human Genetics* 59:501-509.

Howell N, Smejkal CB, Mackey DA, Chinnery PF, Turnbull DM, and Herrnstadt C. 2003. The pedigree rate of sequence divergence in the human mitochondrial genome: there is a difference between phylogenetic and pedigree rates. *American Journal of Human Genetics* 72:659-670.

Huang ZH, Liu NF, Liang W, Zhang YY, Liao XJ, Ruan LZ, and Yang ZS. 2010. Phylogeography of Chinese bamboo partridge, *Bambusicola thoracica thoracica* (Aves: Galliformes) in south China: inference from mitochondrial DNA control-region sequences. *Molecular Phylogenetics and Evolution* 56:273-280.

Huang ZH, Liu NF, Xiao YA, Cheng YL, Mei WF, Wen LY, Zhang LX, and Yu XP. 2009. Phylogenetic relationships of four endemic genera of the Phasianidae in China based on mitochondrial DNA control-region genes. *Molecular Phylogenetics and Evolution* 53:378-383.

Hughes JM. 2006. Phylogeny of the cuckoo genus Coccyzus (Aves: Cuculidae): a test of monophyly. *Systematics and Biodiversity* 4:483-488.

Ingman M, Kaessmann H, Pääbo S, and Gyllensten U. 2000. Mitochondrial genome variation and the origin of modern humans. *Nature* 408:708-713.

Jackson JA, Baker CS, Vant M, Steel DJ, Medrano-Gonzalez L, and Palumbi SR. 2009. Big and slow: phylogenetic estimates of molecular evolution in baleen whales (suborder Mysticeti). *Molecular Biology and Evolution* 26:2427-2440.

Jin YT, and Liu NF. 2010. Phylogeography of *Phrynocephalus erythrurus* from the Qiangtang Plateau of the Tibetan Plateau. *Molecular Phylogenetics and Evolution* 54:933-940.

Johnson KP, and Clayton DH. 2000. A molecular phylogeny of the dove genus *Zenaida*: mitochondrial and nuclear DNA sequences. *The Condor* 102:864-870.

Johnson KP, and Sorenson MD. 1998. Comparing molecular evolution in two mitochondrial protein coding genes (cytochrome b and ND2) in the dabbling ducks (Tribe: Anatini). *Molecular Phylogenetics and Evolution* 10:82-94.

Johnson NK, and Cicero C. 2002. The role of ecologic diversification in sibling speciation of *Empidonax* flycatchers (Tyrannidae): multigene evidence from mtDNA. *Molecular Ecology* 11:2065-2081.

Juan C, Oromi P, and Hewitt GM. 1995. Mitochondrial DNA phylogeny and sequential colonization of Canary Islands by darkling beetles of the genus *Pimelia* (Tenebrionidae). *Proceedings of the Royal Society B - Biological Sciences* 261:173-180.

Kelley J. 2002. The hominoid radiation in Asia. In: Hartwig WC, ed. *The primate fossil record*. Cambridge: Cambridge University Press, 369–384.

Kemp BM, Malhi RS, McDonough J, Bolnick DA, Eshleman JA, Rickards O, Martinez-Labarga C, Johnson JR, Lorenz JG, Dixon EJ, Fifield TE, Heaton TH, Worl R, and Smith DG. 2007. Genetic analysis of early holocene skeletal remains from Alaska and its implications for the settlement of the Americas. *American Journal of Physical Anthropology* 132:605-621.

Kiyoshi T, and Sota T. 2006. Differentiation of the dragonfly genus *Davidius* (Odonata: Gomphidae) in Japan inferred from mitochondrial and nuclear gene genealogies. *Zoological Science* 23:1-8.

Klicka J, Fry AJ, Zink RM, and Thompson CW. 2001. A cytochrome-b perspective on Passerina bunting relationships. *The Auk* 118:611-623.

Kornilios P, Kyriazi P, Poulakakis N, Kumlutas Y, Ilgaz C, Mylonas M, and Lymberakis P. 2010. Phylogeography of the ocellated skink *Chalcides ocellatus* (Squamata, Scincidae), with the use of mtDNA sequences: a hitch-hiker's guide to the Mediterranean. *Molecular Phylogenetics and Evolution* 54:445-456.

Korsten M, Ho SYW, Davison J, Pahn B, Vulla E, Roht M, Tumanov IL, Kojola I, Andersone-Lilley Z, Ozolins J, Pilot M, Mertzanis Y, Giannakopoulos A, Vorobiev AA, Markov NI, Saveljev AP, Lyapunova EA, Abramov AV, Mannil P, Valdmann H, Pazetnov SV, Pazetnov VS, Rokov AM, and Saarma U. 2009. Sudden expansion of a single brown bear maternal lineage across northern continental Eurasia after the last ice age: a general demographic model for mammals? *Molecular Ecology* 18:1963-1979.

Krajewski C, and King DG. 1996. Molecular divergence and phylogeny: rates and patterns of cytochrome b evolution in cranes. *Molecular Biology and Evolution* 13:21-30.

Krijgsman W, Hilgen FJ, Raffi I, Sierro FJ, and Wilson DS. 1999. Chronology, causes and progression of the Messinian salinity crisis. *Nature* 400:652-655.

Kurtén B. 1968. *Pleistocene mammals of Europe*. London, United Kingdom: Weidenfeld and Nicholson.

Kurtén B. 1976. *The Cave Bear Story. Life and Death of a Vanished Animal*. New York, USA: Columbia University Press.

Lambert DM, Ritchie PA, Millar CD, Holland B, Drummond AJ, and Baroni C. 2002. Rates of evolution in ancient DNA from Adélie penguins. *Science* 295:2270-2273.

Lanier HC, and Olson LE. 2009. Inferring divergence times within pikas (*Ochotona* spp.) using mtDNA and relaxed molecular dating techniques. *Molecular Phylogenetics and Evolution* 53:1-12.

Lanyon SM, and Omland KE. 1999. A molecular phylogeny of the blackbirds (Icteridae): Five lineages revealed by cytochrome-b sequence data. *The Auk* 116:629-639.

Lerner HRL, and Mindell DP. 2005. Phylogeny of eagles, Old World vultures, and other Accipitridae based on nuclear and mitochondrial DNA. *Molecular Phylogenetics and Evolution* 37:327-346.

Lindqvist C, Schuster SC, Sun Y, Talbot SL, Qi J, Ratan A, Tomsho LP, Kasson L, Zeyl E, Aars J, Miller W, Ingolfsson O, Bachmann L, and Wiig O. 2010. Complete mitochondrial genome of a Pleistocene jawbone unveils the origin of polar bear. *Proceedings of the National Academy of Sciences of the United States of America* 107:5053-5057.

Lorenzen ED, Nogues-Bravo D, Orlando L, Weinstock J, Binladen J, Marske KA, Ugan A, Borregaard MK, Gilbert MTP, Nielsen R, Ho SYW, Goebel T, Graf KE, Byers D, Stenderup JT, Rasmussen M, Campos PF, Leonard JA, Koepfli KP, Froese D, Zazula G, Stafford TW, Jr., Aaris-Sorensen K, Batra P, Haywood AM, Singarayer JS, Valdes PJ, Boeskorov G, Burns JA, Davydov SP, Haile J, Jenkins DL, Kosintsev P, Kuznetsova T, Lai X, Martin LD, McDonald HG, Mol D, Meldgaard M, Munch K, Stephan E, Sablin M, Sommer RS, Sipko T, Scott E, Suchard MA, Tikhonov A, Willerslev R, Wayne RK, Cooper A, Hofreiter M, Sher A, Shapiro B, Rahbek C, and Willerslev E. 2011. Species-specific responses of Late Quaternary megafauna to climate and humans. *Nature* 479:359-364.

Lovette IJ, and Bermingham E. 2001. Mitochondrial perspective on the phylogenetic relationships of the Parula wood-warblers. *The Auk* 118:211-215.

Lovette IJ, and Hochachka WM. 2006. Simultaneous effects of phylogenetic niche conservatism and competition on avian community structure. *Ecology* 87:S14-S28.

Machado CA, Jousselin E, Kjellberg F, Compton SG, and Herre EA. 2001. Phylogenetic relationships, historical biogeography and character evolution of fig-pollinating wasps. *Proceedings of the Royal Society B - Biological Sciences* 268:685-694.

Madrigal L, Castri L, Melendez-Obando M, Villegas-Palma R, Barrantes R, Raventos H, Pereira R, Luiselli D, Pettener D, and Barbujani G. 2012. High mitochondrial mutation rates estimated from deep-rooting Costa Rican pedigrees. *American Journal of Physical Anthropology* 148:327-333.

Malekian M, Cooper SJB, Norman JA, Christidis L, and Carthew SM. 2010. Molecular systematics and evolutionary origins of the genus Petaurus (Marsupialia: Petauridae) in Australia and New Guinea. *Molecular Phylogenetics and Evolution* 54:122-135.

McDougal I, and Chamalau FG. 1969. Isotopic dating and geomagnetic polarity studies on volcanic rocks from Mauritius, Indian Ocean. *Geological Society of America Bulletin* 80:1419-&.

Melville J, Ritchie EG, Chapple SNJ, Glor RE, and Schulte JA. 2011. Evolutionary origins and diversification of dragon lizards in Australia's tropical savannas. *Molecular Phylogenetics and Evolution* 58:257-270.

Millar CD, Dodd A, Anderson J, Gibb GC, Ritchie PA, Baroni C, Woodhams MD, Hendy MD, and Lambert DM. 2008. Mutation and evolutionary rates in Adélie penguins from the Antarctic. *PLOS Genetics* 4:e1000209.

Miller MJ, Bermingham E, and Ricklefs RE. 2007. Historical biogeography of the new world solitaires (Myadestes spr). *The Auk* 124:868-885.

Mlíkovsky J. 2002. *Cenozoic birds of the world, Part 1: Europe*. Prague: Ninox Press.

Molak M, Lorenzen ED, Shapiro B, and Ho SYW. 2013. Phylogenetic estimation of timescales using ancient DNA: the effects of temporal sampling scheme and uncertainty in sample ages. *Molecular Biology and Evolution* 30:253-262.

Moore WS, Overton LC, and Miglia KJ. 2011. Mitochondrial DNA based phylogeny of the woodpecker genera Colaptes and Piculus, and implications for the history of woodpecker diversification in South America. *Molecular Phylogenetics and Evolution* 58:76-84.

Moum T, Arnason U, and Arnason E. 2002. Mitochondrial DNA sequence evolution and phylogeny of the Atlantic Alcidae, including the extinct great auk (*Pinguinus impennis*). *Molecular Biology and Evolution* 19:1434-1439.

Mumm S, Whyte MP, Thakker RV, Buetow KH, and Schlessinger D. 1997. mtDNA analysis shows common ancestry in two kindreds with X-linked recessive hypoparathyroidism and reveals a heteroplasmic silent mutation. *American Journal of Human Genetics* 60:153-159.

Nabholz B, Glemin S, and Galtier N. 2009. The erratic mitochondrial clock: variations of mutation rate, not population size, affect mtDNA diversity across birds and mammals. *BMC Evolutionary Biology* 9.

Nazari V, and Sperling FAH. 2007. Mitochondrial DNA divergence and phylogeography in western Palaearctic Parnassiinae (Lepidoptera: Papilionidae): how many species are there? *Insect Systematics & Evolution* 38:121-138.

Nunn GB, Cooper J, Jouventin P, Robertson CJR, and Robertson GG. 1996. Evolutionary relationships among extant albatrosses (Procellariiformes: Diomedeidae) established from complete cytochrome-B gene sequences. *The Auk* 113:784-801.

Nunn GB, and Stanley SE. 1998. Body size effects and rates of cytochrome b evolution in tube-nosed seabirds. *Molecular Biology and Evolution* 15:1360-1371.

Olson SL. 1991. The fossil record of the genus *Mycteria* (Ciconiidae) in North-America. *The Condor* 93:1004-1006.

Olson SL, and Rasmussen PD. 2001. Miocene and Pliocene birds from the Lee Creek Mine, North Carolina. In: Ray CE, and Bohaska DJ, eds. *Geology and paleontology of the Lee Creek Mine, North Carolina, Vol III*: Smithsonian Contributions to Paleobiology, 233-365.

Omland KE, Lanyon SM, and Fritz SJ. 1999. A molecular phylogeny of the new world orioles (Icterus): The importance of dense taxon sampling. *Molecular Phylogenetics and Evolution* 12:224-239.

Pacheco MA, Battistuzzi FU, Lentino M, Aguilar RF, Kumar S, and Escalante AA. 2011. Evolution of modern birds revealed by mitogenomics: timing the radiation and origin of major orders. *Molecular Biology and Evolution* 28:1927-1942.

Päckert M, Martens J, Tietze DT, Dietzen C, Wink M, and Kvist L. 2007. Calibration of a molecular clock in tits (Paridae) - Do nucleotide substitution rates of mitochondrial genes deviate from the 2% rule? *Molecular Phylogenetics and Evolution* 44:1-14.

Papadopoulou A, Anastasiou I, and Vogler AP. 2010. Revisiting the insect mitochondrial molecular clock: the mid-Aegean trench calibration. *Molecular Biology and Evolution* 27:1659-1672.

Parsons TJ, and Holland MM. 1998. Mitochondrial mutation rate revisited: hot spots and polymorphism - Response. *Nature Genetics* 18:110-110.

Parsons TJ, Muniec DS, Sullivan K, Woodyatt N, AllistonGreiner R, Wilson MR, Berry DL, Holland KA, Weedn VW, Gill P, and Holland MM. 1997. A high observed substitution rate in the human mitochondrial DNA control region. *Nature Genetics* 15:363-368.

Patane JSL, Weckstein JD, Aleixo A, and Bates JM. 2009. Evolutionary history of Ramphastos toucans: Molecular phylogenetics, temporal diversification, and biogeography. *Molecular Phylogenetics and Evolution* 53:923-934.

Patel S, Weckstein JD, Patane JSL, Bates JM, and Aleixo A. 2011. Temporal and spatial diversification of *Pteroglossus aracaris* (Aves: Ramphastidae) in the neotropics: constant rate of diversification does

not support an increase in radiation during the Pleistocene. *Molecular Phylogenetics and Evolution* 58:105-115.

Paxinos EE, James HF, Olson SL, Ballou JD, Leonard JA, and Fleischer RC. 2002. Prehistoric decline of genetic diversity in the nene. *Science* 296:1827-1827.

Percy DM, Page RDM, and Cronk QCB. 2004. Plant-insect interactions: Double-dating associated insect and plant lineages reveals asynchronous radiations. *Systematic Biology* 53:120-127.

Pereira SL, and Baker AJ. 2005. Multiple gene evidence for parallel evolution and retention of ancestral morphological states in the shanks (Charadriiformes: Scolopacidae). *The Condor* 107:514-526.

Perez-Eman JL. 2005. Molecular phylogenetics and biogeography of the Neotropical redstarts (Myioborus; Aves, Parulinae). *Molecular Phylogenetics and Evolution* 37:511-528.

Pesole G, Gissi C, De Chirico A, and Saccone C. 1999. Nucleotide substitution rate of mammalian mitochondrial genomes. *Journal of Molecular Evolution* 48:427-434.

Pesole G, Sbisa E, Preparata G, and Saccone C. 1992. The Evolution of the Mitochondrial D-Loop Region and the Origin of Modern Man. *Molecular Biology and Evolution* 9:587-598.

Pfeiler E, Bitler BG, Ramsey JM, Palacios-Cardiel C, and Markow TA. 2006. Genetic variation, population structure, and phylogenetic relationships of *Triatoma rubida* and *T. recurva* (Hemiptera: Reduviidae: Triatominae) from the Sonoran Desert, insect vectors of the Chagas' disease parasite *Trypanosoma cruzi*. *Molecular Phylogenetics and Evolution* 41:209-221.

Pinho C, Harris DJ, and Ferrand N. 2007. Contrasting patterns of population subdivision and historical demography in three western Mediterranean lizard species inferred from mitochondrial DNA variation. *Molecular Ecology* 16:1191-1205.

Plazzi F, Ricci A, and Passamonti M. 2011. The mitochondrial genome of *Bacillus* stick insects (Phasmatodea) and the phylogeny of orthopteroid insects. *Molecular Phylogenetics and Evolution* 58:304-316.

Pons J, Ribera I, Bertranpetit J, and Balke M. 2010. Nucleotide substitution rates for the full set of mitochondrial protein-coding genes in Coleoptera. *Molecular Phylogenetics and Evolution* 56:796-807.

Pons J, and Vogler AP. 2005. Complex pattern of coalescence and fast evolution of a mitochondrial rRNA pseudogene in a recent radiation of tiger beetles. *Molecular Biology and Evolution* 22:991-1000.

Pons JM, Hassanin A, and Crochet PA. 2005. Phylogenetic relationships within the Laridae (Charadriiformes: Aves) inferred from mitochondrial markers. *Molecular Phylogenetics and Evolution* 37:686-699.

Prost S, Smirnov N, Fedorov VB, Sommer RS, Stiller M, Nagel D, Knapp M, and Hofreiter M. 2010. Influence of climate warming on arctic mammals? New insights from ancient DNA studies of the collared lemming *Dicrostonyx torquatus*. *PLOS ONE* 5.

Pruser F, and Mossakowski D. 1998. Low substitution rates in mitochondrial DNA in Mediterranean carabid beetles. *Insect Molecular Biology* 7:121-128.

Qu JY, Liu NF, Bao XK, and Wang XL. 2009. Phylogeography of the ring-necked pheasant (*Phasianus colchicus*) in China. *Molecular Phylogenetics and Evolution* 52:125-132.

Qu YH, and Lei FM. 2009. Comparative phylogeography of two endemic birds of the Tibetan plateau, the white-rumped snow finch (*Onychostruthus taczanowskii*) and the Hume's ground tit (*Pseudopodoces humilis*). *Molecular Phylogenetics and Evolution* 51:312-326.

Rohland N, Reich D, Mallick S, Meyer M, Green RE, Georgiadis NJ, Roca AL, and Hofreiter M. 2010. Genomic DNA sequences from mastodon and woolly mammoth reveal deep speciation of forest and savanna elephants. *PLOS Biology* 8:e1000564.

Roman J, and Palumbi SR. 2003. Whales before whaling in the North Atlantic. *Science* 301:508-510.

Rooney AP, Honeycutt RL, and Derr JN. 2001. Historical population size change of bowhead whales inferred from DNA sequence polymorphism data. *Evolution* 55:1678-1685.

Ruiz C, Jordal B, and Serrano J. 2009. Molecular phylogeny of the tribe Sphodrini (Coleoptera: Carabidae) based on mitochondrial and nuclear markers. *Molecular Phylogenetics and Evolution* 50:44-58.

Santos C, Montiel R, Sierra B, Bettencourt C, Fernandez E, Alvarez L, Lima M, Abade A, and Aluja MP. 2005. Understanding differences between phylogenetic and pedigree-derived mtDNA mutation rate: a model using families from the Azores Islands (Portugal). *Molecular Biology and Evolution* 22:1490-1505.

Shapiro B, Drummond AJ, Rambaut A, Wilson MC, Matheus PE, Sher AV, Pybus OG, Gilbert MTP, Barnes I, Binladen J, Willerslev E, Hansen AJ, Baryshnikov GF, Burns JA, Davydov S, Driver JC, Froese DG, Harington CR, Keddie G, Kosintsev P, Kunz ML, Martin LD, Stephenson RO, Storer J, Tedford R, Zimov S, and Cooper A. 2004. Rise and fall of the Beringian steppe bison. *Science* 306:1561-1565.

Shapiro B, Sibthorpe D, Rambaut A, Austin J, Wragg GM, Bininda-Emonds ORP, Lee PLM, and Cooper A. 2002. Flight of the dodo. *Science* 295:1683-1683.

Shapiro LH, Strazanac JS, and Roderick GK. 2006. Molecular phylogeny of Banza (Orthoptera: Tettigoniidae), the endemic katydids of the Hawaiian Archipelago. *Molecular Phylogenetics and Evolution* 41:53-63.

Sheldon FH, Jones CE, and McCracken KG. 2000. Relative patterns and rates of evolution in heron nuclear and mitochondrial DNA. *Molecular Biology and Evolution* 17:437-450.

Shields GF, and Wilson AC. 1987. Calibration of mitochondrial DNA evolution in geese. *Journal of Molecular Evolution* 24:212-217.

Sigurðardóttir S, Helgason A, Gulcher JR, Stefansson K, and Donnelly P. 2000. The mutation rate in the human mtDNA control region. *American Journal of Human Genetics* 66:1599-1609.

Slade RW, Moritz C, Hoelzel AR, and Burton HR. 1998. Molecular population genetics of the southern elephant seal *Mirounga leonina*. *Genetics* 149:1945-1957.

Smith CI, Godsoe WKW, Tank S, Yoder JB, and Pellmyr O. 2008. Distinguishing coevolution from covicariance in an obligate pollination mutualism: asynchronous divergence in Joshua tree and its pollinators. *Evolution* 62:2676-2687.

Soodyall H, Jenkins T, Mukherjee A, DuToit E, Roberts DF, and Stoneking M. 1997. The founding mitochondrial DNA lineages of Tristan da Cunha islanders. *American Journal of Physical Anthropology* 104:157-166.

Sorenson MD, Cooper A, Paxinos EE, Quinn TW, James HF, Olson SL, and Fleischer RC. 1999. Relationships of the extinct moa-nalos, flightless Hawaiian waterfowl, based on ancient DNA. *Proceedings of the Royal Society B - Biological Sciences* 266:2187-2193.

Sota T, and Hayashi M. 2007. Comparative historical biogeography of Plateumaris leaf beetles (Coleoptera : Chrysomelidae) in Japan: interplay between fossil and molecular data. *Journal of Biogeography* 34:977-993.

Speller CF, Kemp BM, Wyatt SD, Monroe C, Lipe WD, Arndt UM, and Yang DY. 2010. Ancient mitochondrial DNA analysis reveals complexity of indigenous North American turkey domestication. *Proceedings of the National Academy of Sciences of the United States of America* 107:2807-2812.

Steiner CC, and Ryder OA. 2011. Molecular phylogeny and evolution of the Perissodactyla. *Zoological Journal of the Linnean Society* 163:1289-1303.

Stoneking M, Sherry ST, Redd AJ, and Vigilant L. 1992. New approaches to dating suggest a recent age for the human mtDNA ancestor. *Philosophical Transactions of the Royal Society of London Series B - Biological Sciences* 337:167-175.

Subramanian S, Denver DR, Millar CD, Heupink T, Aschrafi A, Emslie SD, Baroni C, and Lambert DM. 2009. High mitogenomic evolutionary rates and time dependency. *Trends in Genetics* 25:482-486.

Tamura K, and Nei M. 1993. Estimation of the number of nucleotide substitutions in the control region of mitochondrial DNA in humans and chimpanzees. *Molecular Biology and Evolution* 10:512-526.

Teacher AGF, Thomas JA, and Barnes I. 2011. Modern and ancient red fox (*Vulpes vulpes*) in Europe show an unusual lack of geographical and temporal structuring, and differing responses within the carnivores to historical climatic change. *BMC Evolutionary Biology* 11:214.

Tedford RH, Flynn LJ, Zhanxiang Q, Opdyke ND, and Downs WR. 1991. Yushe Basin, China; paleomagnetically calibrated mammalian biostratigraphic standard from the late Neogene of eastern Asia. *Journal of Vertebrate Paleontology* 11:519-526.

Vigilant L, Stoneking M, Harpending H, Hawkes K, and Wilson AC. 1991. African Populations and the Evolution of Human Mitochondrial-DNA. *Science* 253:1503-1507.

Vrba ES, and Schaller GB. 2000. Phylogeny of Bovidae based on behavior, glands, skulls, and postcrania. In: Vrba ES, and Schaller GB, eds. *Antelopes, Deer, and Relatives Fossil Record, Behavioral Ecology, Systematics, and Conservation*. New Haven, USA: Yale University Press, 203–222.

Wahlberg N. 2006. That awkward age for butterflies: Insights from the age of the butterfly subfamily Nymphalinae (Lepidoptera : Nymphalidae). *Systematic Biology* 55:703-714.

Warren BH, Bermingham E, Bowie RCK, Prys-Jones RP, and Thebaud C. 2003. Molecular phylogeography reveals island colonization history and diversification of western Indian Ocean sunbirds (Nectarinia: Nectariniidae). *Molecular Phylogenetics and Evolution* 29:67-85.

Watanobe T, Ishiguro N, Nakano M, Matsui A, Hongo H, Yamazaki K, and Takahashi O. 2004. Prehistoric Sado Island populations of *Sus scrofa* distinguished from contemporary Japanese wild boar by ancient mitochondrial DNA. *Zoological Science* 21:219-228.

Watanobe T, Ishiguro N, Okumura N, Nakano M, Matsui A, Hongo H, and Ushiro H. 2001. Ancient mitochondrial DNA reveals the origin of *Sus scrofa* from Rebun Island, Japan. *Journal of Molecular Evolution* 52:281-289.

Wayne RK, Geffen E, Girman DJ, Koepfli KP, Lau LM, and Marshall CR. 1997. Molecular systematics of the Canidae. *Systematic Biology* 46:622-653.

Weckstein JD, Zink RM, Blackwell-Rago RC, and Nelson DA. 2001. Anomalous variation in mitochondrial genomes of White-crowned (Zonotrichia leucophrys) and Golden-crowned (Z-atricapilla) sparrows: Pseudogenes, hybridization, or incomplete lineage sorting? *The Auk* 118:231-236.

Weir JT, Bermingham E, Miller MJ, Klicka J, and Gonzalez MA. 2008. Phylogeography of a morphologically diverse Neotropical montane species, the Common Bush-Tanager (*Chlorospingus ophthalmicus*). *Molecular Phylogenetics and Evolution* 47:650-664.

Weir JT, and Schluter D. 2008. Calibrating the avian molecular clock. *Molecular Ecology* 17:2321-2328.

Wilkinson HE. 1969. Description of an upper Miocene albatross from Beaumaris, Victoria, Australia, and a review of fossil Diomedeidae. *Memoirs of the National Museum of Victoria* 29:41-51.

Willerslev E, Gilbert MTP, Binladen J, Ho SYW, Campos PF, Ratan A, Tomsho LP, da Fonseca RR, Sher A, Kuznetsova TV, Nowak-Kemp M, Roth TL, Miller W, and Schuster SC. 2009. Analysis of complete mitochondrial genomes from extinct and extant rhinoceroses reveals lack of phylogenetic resolution. *BMC Evolutionary Biology* 9:95.

Wilting A, Christiansen P, Kitchener AC, Kemp YJM, Ambu L, and Fickel J. 2011. Geographical variation in and evolutionary history of the Sunda clouded leopard (*Neofelis diardi*) (Mammalia: Carnivora: Felidae) with the description of a new subspecies from Borneo. *Molecular Phylogenetics and Evolution* 58:317-328.

Winker K, and Pruett CL. 2006. Seasonal migration, speciation, and morphological convergence in the genus *Catharus* (Turdidae). *The Auk* 123:1052-1068.

Wüster W, Salomão M, Quijada-Mascareñas JA, Thorpe R, and BBBSP. 2002. Origin and evolution of the South American pitviper fauna: evidence from mitochondrial DNA sequence analysis. In: Schuett G, Höggren M, Douglas M, and Greene H, eds. *Biology of the vipers*. Eagle Mountain, Utah: Eagle Mountain Publishing, 111-128.

Yi S, Ellsworth DL, and Li WH. 2002. Slow molecular clocks in Old World monkeys, apes, and humans. *Molecular Biology and Evolution* 19:2191-2198.

Zakharov EV, Caterino MS, and Sperling FAH. 2004. Molecular phylogeny, historical biogeography, and divergence time estimates for swallowtail butterflies of the genus *Papilio* (Lepidoptera: Papilionidae). *Systematic Biology* 53:193-215.

Zamudio KR, and Greene HW. 1997. Phylogeography of the bushmaster (*Lachesis muta*: Viperidae): implications for neotropical biogeography, systematics, and conservation. *Biological Journal of the Linnean Society* 62:421-442.

Zhao Q, Liu HX, Luo LG, and Ji XA. 2011. Comparative population genetics and phylogeography of two lacertid lizards (*Eremias argus* and *E. brenchleyi*) from China. *Molecular Phylogenetics and Evolution* 58:478-491.

Zink RM, and Blackwell RC. 1998. Molecular systematics of the Scaled Quail complex (genus Callipepla). *The Auk* 115:394-403.
