# Supplementary material for: Prolonged decay of molecular rate estimates for metazoan mitochondrial DNA
Source: PeerJ. 2015 Mar 5;3:e821. doi: 10.7717/peerj.821 (PMC4358697; doi:10.7717/peerj.821)
Supplement: Table S2 — Novel rate estimates and re-estimates obtained in this study. Analyses were done using either the ages of the ancient DNA sequences as calibrations, or by including the sister species and using fossil evidence to calibrate the divergence between the two species. Information about the used data set, sister species, details of fossil calibrations and the population model used in Bayesian phylogenetic inference are provided. Substitution rate estimate and calibration time for each data set were, along with previously published ones (Table S1), included in the meta-analysis. Cal, calibration. [file peerj-03-821-s004.docx]

| **Species** | **Source** | **Calibration type** | **Marker** | **Number of samples (ancient + modern + sister species)** | **Time span covered by DNA samples (years)** | **Alignment length (bp)** | **Sister species**  **with GenBank accession number** | **Divergence time** | **Calibration distribution for the age of the root** | **Best-fitting substitution model** | **Population model** | **Substitution rate estimate and 95% credibility interval**  **(substitutions/site/year)** | **Calibration time (years)** |
| --- | --- | --- | --- | --- | --- | --- | --- | --- | --- | --- | --- | --- | --- |
| Adelie penguin (*Pygoscelis adeliae*) | Lambert et al. 2002 | aDNA | d-loop  (non-coding) | 96 + 380 + 0 | 0 – 6,424 | 347 | n/a | n/a | n/a | TVM+G | constant | 2.31×10^-6^  (1.59×10^-5^ – 3.06×10^-6^) | 6.42×10^3^ |
| Turkey (*Meleagris gallopavo gallopavo*) | Speller et al. 2010 | aDNA | d-loop  (non-coding) | 136 + 12 + 0 | 0 – 1,250 | 438 | n/a | n/a | n/a | HKY | constant | 1.71×10^-6^  (7.27×10^-7^ – 2.80×10^-6^) | 1.25×10^3^ |
| Arctic fox (*Vulpes lagopus*) | aDNA: Dalén *et al.* 2007;  cal: Flynn & Galliano 1982, Wayne et al. 1997 | fossil | d-loop  (non-coding) | 8 + 41 +1 | 0 – 16,000 | 294 | Wolf  (*Canis lupus lupus)*  AM711902 | min 9 Mya (oldest *Vulpes* genus fossil);  max 52 Mya (caniform/feliform divergence) | exponential: offset = 9 Mya, 97.5% = 52 Mya | HKY+G | skyride | 3.25×10^-9^  (6.65×10^-10^ – 8.41×10^-9^) | 5.20×10^7^ |
| Aurochs  (*Bos primigenius*) | Edwards et al. 2007 | aDNA | d-loop  (non-coding) | 41 + 0 | 2,000 – 12,300 | 360 | n/a | n/a | n/a | HKY | constant | 8.19×10^-7^  (3.33×10^-7^ – 1.42×10^-6^) | 1.23×10^4^ |
| Aurochs  (*Bos primigenius*) | aDNA: Edwards et al. 2007;  cal: Tedford et al. 1991, Barry at al. 2002 after Ho et al. 2008 | fossil | d-loop  (non-coding) | 41 + 0 + 1 | 2,000 – 12,300 | 362 | Bison  (*Bison bison)*  NC012346 | min 2 Mya (oldest bison fossil);  max 8.9 Mya (stem of bovines) | exponential: offset = 2 Mya, 95% = 8.9 Mya | HKY+G | constant | 5.66×10^-8^  (1.56×10^-8^ – 1.10×10^-7^) | 8.90×10^6^ |
| Bison  (*Bison priscus*) | aDNA: Shapiro *et al*. 2004;  cal: Tedford et al. 1991, Barry at al. 2002 after Ho et al. 2008 | fossil | d-loop  (non-coding) | 160+22+1 | 0 – 60,400 | 613 | Yak  (*Bos grunniens)*  GQ464290 | min 2 Mya (oldest bison fossil);  max 8.9 Mya (stem of bovines) | exponential: offset=2 Mya, 95% = 8.9 Mya | TrN+G | constant | 2.11×10^-7^  (1.41×10^-7^ – 2.86×10^-7^) | 8.90×10^6^ |
| Bowhead whale (*Balaena mysticetus*) | Foote et al. 2013 | aDNA | d-loop  (non-coding) | 114 + 69 + 0 | 0 – 51,000 | 252 | n/a | n/a | n/a | HKY+G | constant | 3.64×10^-7^  (1.65×10^-7^ – 6.12×10^-7^) | 5.10×10^4^ |
| Brown bear (*Ursus arctos*) | aDNA: Korsten *et al*. 2009; Lindqvist *et al*. 2010;  cal: Kurtén 1968, 1976; Hofreiter *et al.* 2002 after Korsten *et al.* 2009 | fossil | d-loop  (non-coding) | 47+66+1 | 0 – 120,000 | 193 | Cave bear  (*Ursus spelaeus)*  EU327344 | Based on fossil evidence for their supposed common ancestor, the Etruscan bear (*Ursus etruscus*) | normal: mean 1.45 Mya, 2.5% = 1.2 Mya, 97.5% = 1.7 Mya | K80+G | constant | 3.92×10^-7^  (2.15×10^-7^ – 6.13×10^-7^) | 1.45×10^6^ |
| Cave lion (*Panthera leo spelaea*) | Barnett et al. 2009 | fossil | d-loop  (non-coding) | 23+0+1 | 11,925 – 58,200 | 216 | Lion  (*Panthera leo leo)*  DQ899919 | based on first fossil appearance of *Panthera leo fossilis* – 650 kya | normal: mean 550 kya ± 25 kya | HKY | constant | 7.73×10^-8^  (4.23×10^-8^ – 1.18×10^-7^) | 5.50×10^5^ |
| Cow  (*Bos taurus*) | Bollongino et al. 2006 | aDNA | d-loop  (non-coding) | 36+91+0 | 0 – 8,065 | 410 | n/a | n/a | n/a | HKY+G | skyride | 6.72×10^-7^  (4.53×10^-7^ – 9.29×10^-7^) | 8.07×10^3^ |
| Cow  (*Bos taurus*) | aDNA: Bollongino et al. 2006;  cal: Tedford et al. 1991, Barry at al. 2002 after Ho et al. 2008 | fossil | d-loop  (non-coding) | 36+91+1 | 0 – 8,065 | 412 | Bison  (*Bison bison)*  NC012346 | min 2 Mya (oldest bison fossil);  max 8.9 Mya (stem of bovines) | exponential: offset = 2 Mya, 95% = 8.9 Mya | HKY+G | constant | 1.02×10^-7^  (4.71×10^-8^ – 1.80×10^-7^) | 8.90×10^6^ |
| Horse  (*Equus ferus*) | aDNA: Lorenzen et al 2011;  cal: Steiner & Ryder 2011 | fossil | d-loop  (non-coding) | 128+0+1 | 2,220 – 43,900 | 349 | Zebra  (*Equus zebra)*  AY651956 | radiation of modern horses 2-4 Mya | uniform: 2-4 Mya | HKY+G | constant | 2.09×10^-7^  (1.21×10^-7^ – 3.22×10^-7^) | 4.00×10^6^ |
| Human  (*Homo sapiens*) | Fu et al. 2013 | aDNA | d-loop  (non-coding) | 10+54+0 | 0 – 39,475 | 1118 | n/a | n/a | n/a | HKY+G | constant | 1.56×10^-7^  (9.27×10^-8^ – 2.33×10^-7^) | 4.00×10^4^ |
| Human  (*Homo sapiens*) | aDNA: Fu et al. 2013;  cal: Yi *et al.* 2002, Ho *et al*. 2005 | fossil | d-loop  (non-coding) | 10+54+1 | 0 – 39,475 | 1124 | Chimpanzee  (*Pan troglodytes)*  X93335 | Based on Sahelanthropus fossil | normal: mean = 6 Mya, 2.5% = 4.5 Mya, 97.5% = 7.5 Mya | HKY+G | constant | 8.47×10^-8^  (5.18×10^-8^ – 1.31×10^-7^) | 6.00×10^6^ |
| Human  (*Homo sapiens*) | DNA: Ingman et al. 2000; cal: Kelley 2002 | fossil | d-loop  (non-coding) | 0+53+1 | 0 | 1124 | Orangutan (*Pongo pygmaeus*)  NC001646 | *Homo*-*Pongo* lineage divergence based on *Sivapithecus* fossil | uniform: 8.5-12.5 Mya | HKY+G | constant | 5.11×10^-8^  (2.63×10^-8^ – 8.76×10^-8^) | 1.25×10^7^ |
| Lemming (*Dicrostonyx torquatus*) | Prost et al. 2010 | aDNA | cyt b  (coding) | 66+10+0 | 0 – 21910 | 282 | n/a | n/a | n/a | K80 | constant | 1.62×10^-7^  (6.54×10^-8^ – 2.81×10^-7^) | 2.19×10^4^ |
| Lemming (*Dicrostonyx torquatus*) | Prost et al. 2010 | aDNA | d-loop  (non-coding) | 67+10+0 | 0 – 21910 | 426 | n/a | n/a | n/a | F81+G | constant | 5.24×10^-8^  (8.64×10^-9^ – 1.14×10^-7^) | 2.19×10^4^ |
| Muskox (*Ovibos moschatus*) | aDNA: Campos *et al*. 2010b; cal: Vrba & Schaller 2000, paleodb.org | fossil | d-loop  (non-coding) | 121+4+1 | 0 – 42,550 | 689 | Bison  (*Bison bison)*  NC012346 | min 11.61 Mya (first Bovinae fossil);  max 20 Mya (first appearance of Bovids) | uniform: 11.61-20 Mya | HKY+G | constant | 5.19×10^-8^  (4.19×10^-8^ – 6.48×10^-8^) | 2.00×10^7^ |
| Red fox  (*Vulpes vulpes* ) | Teacher et al. 2011 | aDNA | cyt b  (coding) | 20+301+0 | 0 – 30,000 | 201 | n/a | n/a | n/a | HKY | constant | 1.64×10^-7^  (5.10×10^-8^ – 3.38×10^-7^) | 3.00×10^4^ |
| Red fox (*Vulpes vulpes* ) | aDNA: Teacher et al. 2011;  cal: Flynn & Galliano 1982, Wayne et al. 1997 | fossil | cyt b  (coding) | 20+301+1 | 0 – 30,000 | 202 | Wolf  (*Canis lupus lupus)*  AM711902 | min 9 Mya (oldest *Vulpes* genus fossil);  max 52 Mya (caniform/feliform divergence) | exponential: offset = 9 Mya, 97.5% = 52 Mya | HKY | constant | 6.85×10^-9^  (3.16×10^-9^ – 1.07×10^-8^) | 5.20×10^4^ |
| Red fox (*Vulpes vulpes* ) | Teacher et al. 2011 | aDNA | d-loop  (non-coding) | 20+301+0 | 0 – 30,000 | 193 | n/a | n/a | n/a | TIM+G | constant | 6.18×10^-7^  (2.94×10^-7^ – 1.04×10^-6^) | 3.00×10^4^ |
| Red fox (*Vulpes vulpes* ) | aDNA: Teacher et al. 2011;  cal: Flynn & Galliano 1982, Wayne et al. 1997 | fossil | d-loop  (non-coding) | 20+301+1 | 0 – 30,000 | 196 | Wolf  (*Canis lupus lupus)*  AM711902 | min 9 Mya (oldest *Vulpes* genus fossil);  max 52 Mya (caniform/feliform divergence) | exponential: offset = 9 Mya, 97.5% = 52 Mya | TIM+G | skyride | 9.29×10^-10^  (1.89×10^-10^ – 2.25×10^-9^) | 5.20×10^7^ |
| Woolly mammoth (*Mammuthus primigenius*) | Debruyne et al. 2008 | aDNA | cyt b, trnT, trnP  (coding) | 103+0+0 | 5,007 – 55,082 | 416 | n/a | n/a | n/a | HKY | constant | 6.30×10^-8^  (2.72×10^-8^ – 1.12×10^-7^) | 5.51×10^4^ |
| Woolly mammoth (*Mammuthus primigenius*) | aDNA: Debruyne et al. 2008;  cal: Rohland et al. 2010 | fossil | cyt b, trnT, trnP  (coding) | 103+0+1 | 5,007 – 55,082 | 416 | Asian elephant  (*Elephas maximus)*  NC005129 | min 3 Mya (earliest mammoth fossil);  max 8.5 Mya (possibly ancestral *Elephas nawataensis*) | uniform: 3-8.5 Mya | HKY | constant | 6.42×10^-10^  (2.96×10^-10^ – 1.06×10^-9^) | 8.50×10^6^ |
| Woolly mammoth (*Mammuthus primigenius*) | aDNA: Debruyne et al. 2008; cal: Rohland et al. 2010 | fossil | cyt b, trnT, trnP  (coding) | 103+0+1 | 5,007 – 55,082 | 416 | African elephant  (*Loxodonta africana)*  NC000934 | min 4.2 Mya (most ancient representative of *Elephas*);  max 9 Mya (assuming trichotomy) | uniform: 4.2-9 Mya | HKY | constant | 4.34×10^-10^  (2.36×10^-10^ – 6.83×10^-10^) | 9.00×10^6^ |
| Woolly mammoth (*Mammuthus primigenius*) | aDNA: Debruyne et al. 2008; cal: Rohland et al. 2010 | fossil | cyt b, trnT, trnP  (coding) | 103+0+1 | 5,007 – 55,082 | 416 | Mastodon  (*Mammut americanum)*  NC009574 | min 24 Mya (first mammutid fossil);  max 30 Mya (common ancestor fossil) | uniform: 24-30 Mya | HKY | constant | 1.78×10^-10^  (1.29×10^-10^ – 2.35×10^-10^) | 3.00×10^7^ |
| Woolly mammoth (*Mammuthus primigenius*) | Debruyne et al. 2008 | aDNA | d-loop  (non-coding) | 103+0+0 | 5,007 – 55,082 | 297 | n/a | n/a | n/a | HKY+G | constant | 1.99×10^-7^  (8.14×10^-8^ – 4.04×10^-7^)^d^ | 5.51×10^4^ |
| Woolly mammoth (*Mammuthus primigenius*) | aDNA: Debruyne et al. 2008; cal: Rohland et al. 2010 | fossil | d-loop  (non-coding) | 103+0+1 | 5,007 – 55,082 | 297 | Asian elephant  (*Elephas maximus)*  NC005129 | min 3 Mya (earliest mammoth fossil);  max 8.5 Mya (possibly ancestral *Elephas nawataensis*) | uniform: 3-8.5 Mya | HKY+G | constant | 2.40×10^-8^  (4.07×10^-9^ – 5.88×10^-8^) | 8.50×10^6^ |
| Woolly mammoth (*Mammuthus primigenius*) | aDNA: Debruyne et al. 2008; cal: Rohland et al. 2010 | fossil | d-loop  (non-coding) | 103+0+1 | 5,007 – 55,082 | 297 | African elephant  (*Loxodonta africana)*  NC000934 | min 4.2 Mya (most ancient representative of *Elephas*);  max 9 Mya (assuming trichotomy) | uniform: 4.2-9 Mya | HKY+G | constant | 2.07×10^-8^  (4.40×10^-9^ – 5.28×10^-8^) | 9.00×10^6^ |
| Woolly mammoth (*Mammuthus primigenius*) | aDNA: Debruyne et al. 2008; cal: Rohland et al. 2010 | fossil | d-loop  (non-coding) | 103+0+1 | 5,007 – 55,082 | 297 | Mastodon  (*Mammut americanum)*  NC009574 | min 24 Mya (first mammutid fossil);  max 30 Mya (common ancestor fossil) | uniform: 24-30 Mya | HKY+G | constant | 5.15×10^-10^  (2.98×10^-10^ – 8.70×10^-10^) | 3.00×10^7^ |
| Woolly rhinoceros (*Coelodonta antiquitatis*) | aDNA: Lorenzen et al 2011; cal: Willerslev et al. 2009; Carroll 1988 | fossil | d-loop  (non-coding) | 55+0+1 | 12,460 – 43,850 | 547 | Sumatran rhinoceros  (*Dicerorhinus sumatrensis)*  FJ905816 | min 16 Mya (*C.antiquitatis* fossils - lower Miocene)  no more than 56 Mya (rhinoceroses fossils - begining late Eocene) | exponential: offset = 16 Mya, 95% = 56 Mya | TrN+G | constant | 1.38×10^-7^  (3.60×10^-8^ – 2.54×10^-7^) | 5.60×10^7^ |
