# Supplementary material for: Prolonged decay of molecular rate estimates for metazoan mitochondrial DNA
Source: PeerJ. 2015 Mar 5;3:e821. doi: 10.7717/peerj.821 (PMC4358697; doi:10.7717/peerj.821)
Supplement: Table S3 — Linear-regression analysis of log-transformed rate estimates against the log-transformed calibration times that were used to estimate the rates for data subsets. [file peerj-03-821-s005.docx]

|  | **Data subset** | **All** | **Insects** | **Reptiles** | **Birds** | **Mammals** | **Humans** |
| --- | --- | --- | --- | --- | --- | --- | --- |
| **Coding markers** | **N** | 182 | 33 | 18 | 101 | 30 | n/a |
|  | **R^2^** | 0.24 | 0.38 | 0.32 | 0.08 | 0.29 | n/a |
|  | **P-value** | 1.58×10^-12^  *** | 1.34×10^-4^  *** | 1.40×10^-2^  * | 4.87×10^-3^  ** | 2.03×10^-3^  ** | n/a |
|  | **Slope** | -0.28 | -0.25 | -0.24 | -0.19 | -0.33 | n/a |
|  | **Slope std err** | 0.04 | 0.06 | 0.09 | 0.06 | 0.10 | n/a |
|  | **Non-SSC**  **Randomization^a^** | <0.0001  *** | <0.0001  *** | <0.0001  *** | <0.0001  *** | <0.0001  *** | n/a |
|  | **Non-SSC**  **H_0_: s=-1^b^** | 1.97×10^-47^  *** | 3.55×10^-14^  *** | 1.59×10^-7^  *** | 2.12×10^-22^  *** | 2.13×10^-7^  *** | n/a |
| **Non-coding markers** | **N** | 57 | 0 | 2 | 9 | 46 | 26 |
|  | **R^2^** | 0.60 | n/a | n/a | 0.67 | 0.57 | 0.69 |
|  | **P-value** | 1.38×10^-12^  *** | n/a | n/a | 6.60×10^-3^  * | 1.50×10^-9^  *** | 1.51×10^-7^  *** |
|  | **Slope** | -0.43 | n/a | n/a | -0.38 | -0.44 | -0.24 |
|  | **Slope std err** | 0.05 | n/a | n/a | 0.10 | 0.06 | 0.03 |
|  | **Non-SSC**  **Randomization^a^** | <0.0001  *** | n/a | n/a | 0.0001  *** | <0.0001  *** | <0.0001  *** |
|  | **Non-SSC**  **H_0_: s=-1^b^** | 3.88×10^-17^  *** | n/a | n/a | 4.87×10^-4^  *** | 3.09×10^-12^  *** | 5.42×10^-18^  *** |

Non-SSC – results of tests against spurious self-correlation: ^a^ using randomized genetic distances to create 10,000 new regressions and inspecting whether the original slope estimate falls within the distribution of slopes estimated from the randomized data or ^b^using slope s=-1 (average slope for all regressions with randomized genetic distance) as null hypothesis for regression; * p<0.05, ** p<0.005, *** p<0.0005.
