# Supplementary material for: Prolonged decay of molecular rate estimates for metazoan mitochondrial DNA
Source: PeerJ. 2015 Mar 5;3:e821. doi: 10.7717/peerj.821 (PMC4358697; doi:10.7717/peerj.821)
Supplement: Table S4 — Linear-regression analysis of log-transformed rate estimates against the log-transformed calibration times that were used to estimate the rates. Analyses were done for time-slice subsets of data using a sliding window with a width of four orders of magnitude or using a shrinking window for the oldest calibration times. [file peerj-03-821-s006.docx]

|  | **Time-slice subset** | **10^0^-10^4^ years** | **10^1^-10^5^ years** | **10^2^-10^6^ years** | **10^3^-10^7^ years** | **10^4^-10^8^ years** | **10^5^-10^9^ years** | **10^6^-10^9^ years** | **10^7^-10^9^ years** | **10^8^-10^9^ years** |
| --- | --- | --- | --- | --- | --- | --- | --- | --- | --- | --- |
| **Coding markers** | **N** | 1 | 9 | 20 | 132 | 176 | 173 | 162 | 50 | 5 |
|  | **R^2^** | 0 | 0.05 | 0.24 | 0.31 | 0.23 | 0.09 | 0.04 | 0.11 | 0.04 |
|  | **P-value** | n/a | 5.67×10^-1^ | 2.67×10^-2^  * | 3.43×10^-12^  *** | 1.48×10^-11^  *** | 4.79×10^-5^  *** | 1.26×10^-2^  * | 1.76×10^-2^  * | 7.6×10^-1^ |
|  | **Slope** | n/a | -0.33 | -0.34 | -0.37 | -0.29 | -0.20 | -0.15 | -0.34 | 0.36 |
|  | **Slope std err** | n/a | 0.55 | 0.14 | 0.05 | 0.04 | 0.05 | 0.06 | 0.14 | 1.07 |
|  | **Non-SSC**  **Randomization^a^** | - | 0.137 | <0.0001  *** | <0.0001  *** | <0.0001  *** | <0.0001  *** | <0.0001  *** | <0.0001  *** | 0.1387 |
|  | **Non-SSC**  **H_0_: s=-1^b^** | n/a | 2.60×10^-1^ | 1.82×10^-4^ *** | 7.12×10^-26^  *** | 9.12×10^-40^  *** | 1.11×10^-36^  *** | 6.96×10^-31^  *** | 1.52×10^-5^  *** | 2.94×10^-1^ |
| **Non-coding markers** | **N** | 8 | 25 | 27 | 43 | 48 | 31 | 29 | 12 | 1 |
|  | **R^2^** | 0.12 | 0.27 | 0.33 | 0.62 | 0.55 | 0.27 | 0.17 | 0.16 | 0 |
|  | **P-value** | 4.09×10^-1^ | 7.41×10^-3^  * | 1.80×10^-3^  ** | 4.47×10^-10^  *** | 1.41×10^-9^  *** | 2.58×10^-3^  ** | 2.55×10^-2^  * | 2.00×10^-1^ | n/a |
|  | **Slope** | 0.08 | -0.34 | -0.36 | -0.48 | -0.49 | -0.58 | -0.53 | -0.89 | n/a |
|  | **Slope std err** | 0.10 | 0.12 | 0.10 | 0.06 | 0.06 | 0.17 | 0.22 | 0.65 | n/a |
|  | **Non-SSC**  **Randomization^a^** | 0.0023  ** | 0.0001  *** | <0.0001  *** | <0.0001  *** | <0.0001  *** | 0.0098  ** | 0.02  * | 0.4305 | - |
|  | **Non-SSC**  **H_0_: s=-1^b^** | 2.79×10^-5^  *** | 9.61×10^-6^  *** | 1.31×10^-6^  *** | 8.46×10^-11^  *** | 4.31×10^-10^  *** | 2.20×10^-2^  * | 4.39×10^-2^  * | 8.64×10^-1^ | n/a |

^a^Non-SSC – results of tests against spurious self-correlation using randomized genetic distances to create 10,000 new regressions and inspecting whether the original estimate of the slope falls within the distribution of slopes estimated from the randomized data

^b^Non-SSC – results of tests against spurious self-correlation using slope s=-1 (average slope for all regressions with randomized genetic distance) as null hypothesis for regression;

* p<0.05, ** p<0.005, *** p<0.0005.
