# Supplementary material for: Prolonged decay of molecular rate estimates for metazoan mitochondrial DNA
Source: PeerJ. 2015 Mar 5;3:e821. doi: 10.7717/peerj.821 (PMC4358697; doi:10.7717/peerj.821)
Supplement: Supplemental Information 7 [file peerj-03-821-s007.doc]

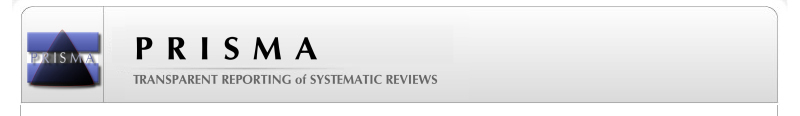
**PRISMA 2009 Flow Diagram**

**Screening**

**Included**

**Eligibility**

**Identification**

Records identified through database searching
(n = 283)

Additional records identified through other sources

(novel estimates and reestimates obtained in this study)
(n = 31)

Records after duplicates removed
(n = 239)

Records screened
(n = 239)

Records excluded
(n = 0)

Full-text articles assessed for eligibility
(n = 239)

Full-text articles excluded, with reasons
(n = 0)

Studies included in qualitative synthesis
(n = 239)

Studies included in quantitative synthesis (meta-analysis)
(n = 239)
